# Supplementary figures and images for: The biphasic and age-dependent impact of klotho on hallmarks of aging and skeletal muscle function
Source: eLife. 2021 Apr 20;10:e61138. doi: 10.7554/eLife.61138 (PMC8118657; doi:10.7554/eLife.61138)

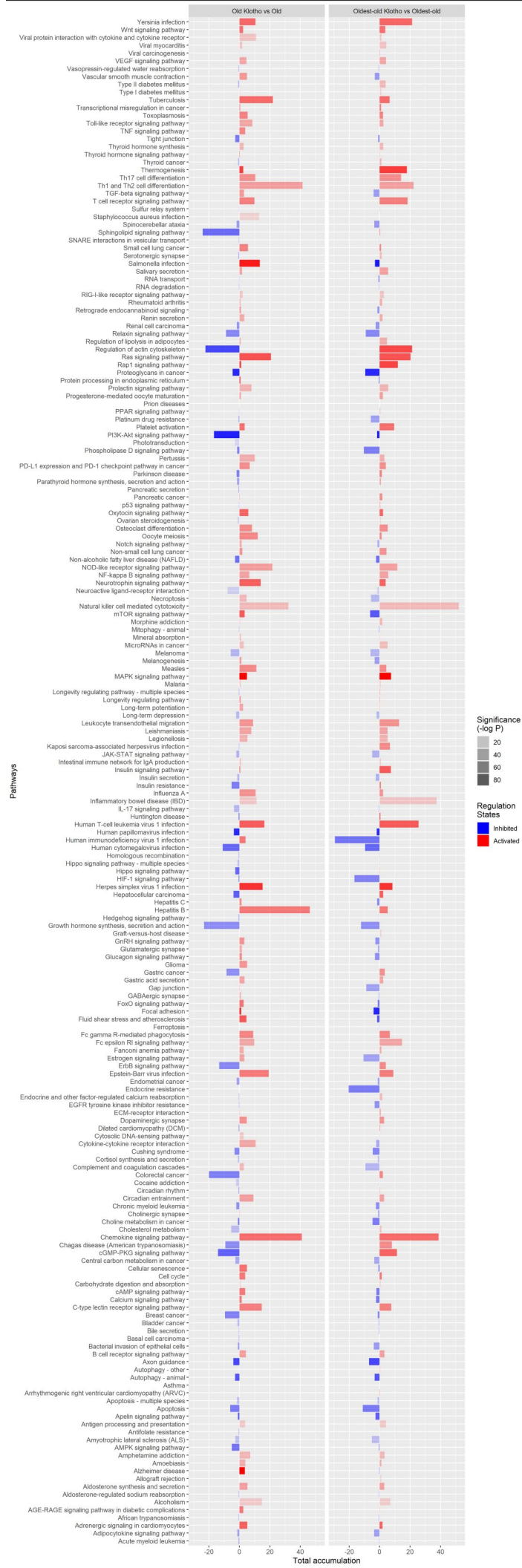

Pathways

Significance  
(-log P)

Regulation  
States

Inhibited  
Activated

Total accumulation

Supplement: Supplementary file 1. [file elife-61138-supp1.pdf]
