# Supplementary material for: The biphasic and age-dependent impact of klotho on hallmarks of aging and skeletal muscle function
Source: eLife. 2021 Apr 20;10:e61138. doi: 10.7554/eLife.61138 (PMC8118657; doi:10.7554/eLife.61138)
Supplement: Supplementary file 2. [file elife-61138-supp2.docx]

**Supplemental Table 1.** Search terms used to identify genes that belong to each hallmark

| **Hallmarks** | **Search terms** |
| --- | --- |
| 1. Genomic instability | “DNA stability” (4 genes)  “nuclear lamina” (13 genes)  “Mitochondrial DNA” (26 genes)  “DNA repair” (495 genes) |
| 1. Telomere attrition | “telomere” (193 genes)  “telomerase” (96 genes)  “ROS” (430 genes) |
| 1. Epigenetic alteration | “histone modification” (517 genes)  “post-translational modification” (25 genes)  “chromatin remodeling” (181 genes)  “DNA methylation” (106 genes) |
| 1. Loss of proteostasis | “proteolytic” (66 genes)  “autophagy” (403 genes)  “proteasome” (943 genes)  “heat-shock protein” (251 genes)  “ubiquitin” (1324 genes)  “protein translation” (617 genes)  “unfolded protein response” (67 genes) |
| 1. Nutrient sensing (NS) deregulation | “insulin” (572 genes)  “growth factor” (972 genes)  “mTOR” (14 genes)  “AMPK” (34genes)  “identical protein binding” AND “transcription factor binding” (168 genes)  “NAD” (469 genes)  “glucose metabolism” (203 genes)  “oxidative metabolism” (286 genes) |
| 1. Mitochondrial dysfunction | “mitochondria” (1961 genes)  “ROS” (430 genes)  “mitophagy” (67 genes) |
| 1. Cellular senescence | “senescence” OR “senescent” (74 genes)  “mitogen” (841 genes) |
| 1. Altered intracellular communication | “extracellular matrix” (829 genes)  “extracellular vesicle” (105 genes)  “paracrine” (221 genes)  “interferon” (1198 genes) |
| 1. Stem cell exhaustion | This hallmark was not included in our final analysis because all the keywords for stem cell exhaustion were already covered under in one of the other hallmarks. This would have resulted in no unique genes for “stem cell exhaustion”. |

**Supplemental Table 2**

1. Network properties of hallmarks of aging genes corresponding to Figure 3E.

| **Sample name** | **Age (months)** | **Number of nodes** | **Number of edges** | **Spatial entropy** | **Spatial entropy per node** | **Link density** | **Unconnected nodes** | **Connected components** |
| --- | --- | --- | --- | --- | --- | --- | --- | --- |
| Young GFP 1 | 3-6 | 6586 | 1093370 | 3420817.541 | 519.40746 | 0.049161993 | 17313 | 1 |
| Young GFP 2 | 3-6 | 6850 | 1144332 | 3610628.962 | 527.09912 | 0.048496767 | 17497 | 1 |
| Young GFP 3 | 3-6 | 6669 | 1108547 | 3478258.915 | 521.55629 | 0.049517495 | 17218 | 1 |
| Young GFP 4 | 3-6 | 6721 | 1109599 | 3488706.71 | 519.07554 | 0.048758852 | 17363 | 1 |
| Old GFP 1 | 21-24 | 6826 | 1146699 | 3615282.605 | 529.63413 | 0.049306835 | 17340 | 1 |
| Old GFP 2 | 21-24 | 6740 | 1121812 | 3527757.61 | 523.40617 | 0.048529841 | 17439 | 1 |
| Old GFP 3 | 21-24 | 6835 | 1143480 | 3607466.1 | 527.79314 | 0.049206548 | 17411 | 1 |
| Old GFP 4 | 21-24 | 6870 | 1157307 | 3657979.725 | 532.45702 | 0.049210541 | 17315 | 1 |
| Oldest-old GFP 1 | 27-29 | 6832 | 1141501 | 3594860.777 | 526.17986 | 0.050099944 | 17110 | 1 |
| Oldest-old GFP 2 | 27-29 | 6766 | 1126263 | 3543798.152 | 523.76562 | 0.048551973 | 17438 | 1 |
| Oldest-old GFP 3 | 27-29 | 6756 | 1126918 | 3542554.077 | 524.35673 | 0.048865689 | 17402 | 1 |
| Oldest-old GFP 4 | 27-29 | 6847 | 1144483 | 3611364.377 | 527.43747 | 0.048392291 | 17479 | 1 |

1. Network properties of all genes corresponding to Figure 3-figure supplement 2.

| **Sample Name** | **Age (months)** | **Number of nodes** | **Number of edges** | **Spatial entropy** | **Spatial entropy per node** | **Link density** | **Disconnected nodes** | **Connected components** |
| --- | --- | --- | --- | --- | --- | --- | --- | --- |
| Young 1 | 3-6 | 15013 | 3609879 | 12210640 | 813.3378 | 0.030809 | 39915 | 3 |
| Young 2 | 3-6 | 15877 | 3839524 | 13133705 | 827.2158 | 0.031258 | 39390 | 2 |
| Young 3 | 3-6 | 15174 | 3668801 | 12424426 | 818.797 | 0.030961 | 39502 | 2 |
| Young 4 | 3-6 | 15382 | 3696147 | 12573382 | 817.4088 | 0.030774 | 39821 | 2 |
| Old 1 | 21-24 | 15729 | 3831916 | 13097729 | 832.7121 | 0.030767 | 39866 | 1 |
| Old 2 | 21-24 | 15419 | 3724637 | 12669459 | 821.6784 | 0.030956 | 39723 | 1 |
| Old 3 | 21-24 | 15692 | 3811095 | 12996181 | 828.2042 | 0.031335 | 39204 | 2 |
| Old 4 | 21-24 | 15890 | 3884024 | 13296088 | 836.7582 | 0.030979 | 39670 | 2 |
| Oldest-old 1 | 27-29 | 15794 | 3838052 | 13067923 | 827.3979 | 0.031245 | 38914 | 1 |
| Oldest-old 2 | 27-29 | 15584 | 3759347 | 12797610 | 821.2018 | 0.03187 | 38783 | 2 |
| Oldest-old 3 | 27-29 | 15494 | 3751720 | 12744648 | 822.5538 | 0.030465 | 40075 | 1 |
| Oldest-old 4 | 27-29 | 15811 | 3850652 | 13106988 | 828.9791 | 0.032034 | 38435 | 3 |

1. Network properties of all genes in male mice from Tabula Muris Senis corresponding to Figure 3-figure supplement 4A. One 21-month-old male sample was excluded since the number of nodes was 33% lower than the mean of rest of the samples.

| **Age (months)** | **Age group** | **Number of nodes** | **Number of edges** | **Spatial entropy** | **Spatial entropy per node** | **Link density** | **Disconnected nodes** | **Connected components** |  |  |
| --- | --- | --- | --- | --- | --- | --- | --- | --- | --- | --- |
| 3 | young | 14559 | 3387952 | 11465614 | 787.5276 | 0.026793 | 42994 | 4 |  |  |
| 3 | young | 15468 | 3592703 | 12239849 | 791.3013 | 0.026817 | 42890 | 2 |  |  |
| 3 | young | 13983 | 3276069 | 10966315 | 784.2605 | 0.030408 | 39261 | 3 |  |  |
| 3 | young | 14453 | 3317662 | 11229869 | 776.9922 | 0.027715 | 41836 | 3 |  |  |
| 6 | young | 17275 | 3997571 | 14009211 | 810.9529 | 0.027693 | 42122 | 2 |  |  |
| 6 | young | 17237 | 3983614 | 13942114 | 808.848 | 0.031754 | 37903 | 3 |  |  |
| 12 | middle age | 15122 | 3476544 | 11838456 | 782.8631 | 0.031523 | 38120 | 3 |  |  |
| 12 | middle age | 16666 | 3848774 | 13374760 | 802.5177 | 0.034207 | 36148 | 4 |  |  |
| 12 | middle age | 16900 | 3954508 | 13719058 | 811.7786 | 0.031323 | 38212 | 3 |  |  |
| 12 | middle age | 14387 | 3286120 | 11099213 | 771.4752 | 0.027625 | 42221 | 4 |  |  |
| 15 | middle age | 14591 | 3355386 | 11343917 | 777.4599 | 0.032353 | 37579 | 3 |  |  |
| 15 | middle age | 13629 | 3176745 | 10617214 | 779.0164 | 0.028191 | 41561 | 3 |  |  |
| 15 | middle age | 14668 | 3369343 | 11385343 | 776.2028 | 0.0323 | 37526 | 3 |  |  |
| 21 | old | 16830 | 3912173 | 13641955 | 810.5737 | 0.029102 | 40194 | 3 |  |  |
| 21 | old | 14306 | 3310434 | 11157262 | 779.9009 | 0.026013 | 44217 | 2 |  |  |
| 21 | old | 16433 | 3806154 | 13179220 | 801.9972 | 0.026041 | 43951 | 1 |  |  |
| 24 | old | 14213 | 3262199 | 10973825 | 772.0977 | 0.026462 | 43378 | 1 |  |  |
| 24 | old | 15640 | 3559057 | 12196107 | 779.8023 | 0.030463 | 38730 | 4 |  |  |
| 24 | old | 18154 | 4286274 | 15113149 | 832.4969 | 0.026415 | 43580 | 1 |  |  |
| 27 | oldest-old | 17911 | 4176811 | 14718573 | 821.7617 | 0.031969 | 38029 | 3 |  |  |
| 27 | oldest-old | 17671 | 4131269 | 14483102 | 819.5972 | 0.030034 | 39743 | 3 |  |  |
| 27 | oldest-old | 14921 | 3390838 | 11575733 | 775.8014 | 0.033513 | 36740 | 4 |  |  |
| 27 | oldest-old | 17613 | 4097036 | 14416374 | 818.5076 | 0.031767 | 37868 | 3 |  |  |

1. Network properties of all genes in male rats corresponding to Figure 3-figure supplement 4B.

| **Age (Months)** | **Number of nodes** | **Number of edges** | **Spatial entropy** | **Spatial entropy per node** | **Link density** | **Disconnected nodes** | **Connected components** |
| --- | --- | --- | --- | --- | --- | --- | --- |
| 6 | 13122 | 563895 | 2343752 | 178.6124 | 0.0066 | 7413 | 1 |
| 6 | 13118 | 563795 | 2344953 | 178.7584 | 0.006661 | 7156 | 1 |
| 6 | 13117 | 567724 | 2359640 | 179.8918 | 0.006618 | 7236 | 1 |
| 6 | 13167 | 564397 | 2353041 | 178.7074 | 0.006626 | 7350 | 1 |
| 6 | 13116 | 558939 | 2326483 | 177.3774 | 0.006576 | 7295 | 1 |
| 6 | 13240 | 580126 | 2407841 | 181.8611 | 0.006553 | 7209 | 1 |
| 6 | 13270 | 577400 | 2396372 | 180.5857 | 0.00663 | 7147 | 1 |
| 9 | 13387 | 586156 | 2443015 | 182.4916 | 0.006569 | 7181 | 1 |
| 9 | 13101 | 564327 | 2350052 | 179.3796 | 0.006631 | 7160 | 1 |
| 9 | 12951 | 557857 | 2314684 | 178.7263 | 0.006642 | 7166 | 1 |
| 9 | 13194 | 567405 | 2364313 | 179.1961 | 0.006569 | 7217 | 1 |
| 9 | 13283 | 578029 | 2401261 | 180.777 | 0.006648 | 7282 | 1 |
| 9 | 13080 | 567517 | 2355981 | 180.1209 | 0.006614 | 7219 | 1 |
| 9 | 12920 | 556887 | 2313689 | 179.0781 | 0.006613 | 7148 | 1 |
| 12 | 12923 | 548493 | 2278226 | 176.2924 | 0.006567 | 7310 | 1 |
| 12 | 13098 | 567627 | 2355468 | 179.8342 | 0.006577 | 7296 | 1 |
| 12 | 13210 | 578123 | 2400637 | 181.7288 | 0.006694 | 7229 | 1 |
| 12 | 13161 | 569470 | 2369333 | 180.0268 | 0.006622 | 7203 | 1 |
| 12 | 13032 | 556376 | 2314072 | 177.5685 | 0.006662 | 7116 | 1 |
| 12 | 12784 | 541717 | 2250656 | 176.0526 | 0.006568 | 7211 | 1 |
| 12 | 12938 | 557483 | 2316073 | 179.0132 | 0.006584 | 7216 | 1 |
| 12 | 13340 | 587197 | 2442081 | 183.0646 | 0.006643 | 7222 | 1 |
| 18 | 12876 | 549599 | 2278307 | 176.9422 | 0.006579 | 7258 | 1 |
| 18 | 12881 | 550987 | 2280761 | 177.064 | 0.006616 | 7178 | 1 |
| 18 | 13061 | 560245 | 2324981 | 178.0094 | 0.006605 | 7296 | 1 |
| 18 | 13132 | 573198 | 2377061 | 181.0129 | 0.006594 | 7214 | 1 |
| 18 | 13056 | 563670 | 2338963 | 179.1485 | 0.006522 | 7379 | 1 |
| 18 | 12859 | 546706 | 2267775 | 176.357 | 0.006569 | 7375 | 1 |
| 18 | 13197 | 571819 | 2371600 | 179.7075 | 0.006595 | 7315 | 1 |
| 21 | 13156 | 569125 | 2362845 | 179.6021 | 0.00653 | 7238 | 1 |
| 21 | 12975 | 559109 | 2319647 | 178.7782 | 0.006622 | 7308 | 1 |
| 21 | 13075 | 562772 | 2339438 | 178.9245 | 0.006585 | 7400 | 1 |
| 21 | 13016 | 556352 | 2313043 | 177.7077 | 0.006529 | 7361 | 1 |
| 21 | 12796 | 545375 | 2262126 | 176.7838 | 0.006447 | 7531 | 1 |
| 21 | 12970 | 556943 | 2311659 | 178.2312 | 0.006569 | 7278 | 1 |
| 21 | 13038 | 568948 | 2360061 | 181.014 | 0.006504 | 7401 | 1 |
| 24 | 13123 | 562238 | 2340381 | 178.3419 | 0.006522 | 7380 | 1 |
| 24 | 13153 | 569030 | 2363414 | 179.6863 | 0.006501 | 7392 | 1 |
| 24 | 12973 | 556658 | 2310024 | 178.064 | 0.006553 | 7293 | 1 |
| 24 | 13228 | 577838 | 2394160 | 180.9919 | 0.0066 | 7329 | 1 |
| 24 | 13071 | 563231 | 2338411 | 178.9007 | 0.006511 | 7327 | 1 |
| 24 | 13305 | 581404 | 2410887 | 181.2016 | 0.00655 | 7317 | 1 |
| 24 | 13218 | 576095 | 2389498 | 180.7761 | 0.006499 | 7224 | 1 |
| 24 | 13355 | 581547 | 2421073 | 181.2859 | 0.006619 | 7313 | 1 |
| 27 | 13356 | 587246 | 2442021 | 182.8407 | 0.006558 | 7357 | 1 |
| 27 | 13233 | 571594 | 2369490 | 179.0592 | 0.006673 | 7189 | 1 |
| 27 | 13614 | 597390 | 2485742 | 182.5872 | 0.006576 | 7274 | 1 |
| 27 | 13136 | 566742 | 2360256 | 179.6784 | 0.006652 | 7189 | 1 |
| 27 | 13301 | 575311 | 2394269 | 180.0067 | 0.006519 | 7363 | 1 |
| 27 | 13354 | 581496 | 2426694 | 181.7204 | 0.006553 | 7382 | 1 |
| 27 | 13313 | 576099 | 2394492 | 179.8612 | 0.006635 | 7299 | 1 |
| 27 | 13161 | 573497 | 2373968 | 180.379 | 0.006542 | 7412 | 1 |

1. Network properties of all genes in male humans corresponding to Figure 3-figure supplement 4C.

| **Decade** | **Age** | **Number of nodes** | **Number of edges** | **Spatial entropy** | **Spatial entropy per node** | **Link density** | **Disconnected nodes** | **Connected components** |  |
| --- | --- | --- | --- | --- | --- | --- | --- | --- | --- |
| 20s | 22 | 17940 | 922540 | 3991017 | 222.4647 | 0.005733 | 83409 | 1 |  |
| 20s | 23 | 15971 | 771970 | 3283312 | 205.5796 | 0.006053 | 78697 | 1 |  |
| 20s | 25 | 17799 | 910126 | 3928206 | 220.6981 | 0.005746 | 83137 | 1 |  |
| 20s | 28 | 15256 | 742179 | 3127276 | 204.9866 | 0.006378 | 77486 | 1 |  |
| 30s | 31 | 14339 | 656839 | 2746332 | 191.5288 | 0.006415 | 77535 | 1 |  |
| 30s | 31 | 17401 | 878215 | 3782220 | 217.3565 | 0.00639 | 73863 | 1 |  |
| 30s | 33 | 17935 | 923124 | 3994548 | 222.7236 | 0.00574 | 83369 | 1 |  |
| 30s | 35 | 17896 | 922128 | 3989031 | 222.9007 | 0.005759 | 83335 | 1 |  |
| 30s | 37 | 16045 | 801930 | 3397343 | 211.7384 | 0.00577 | 82782 | 1 |  |
| 30s | 38 | 16307 | 825481 | 3513008 | 215.4294 | 0.006239 | 77188 | 1 |  |
| 30s | 38 | 15199 | 720633 | 3040551 | 200.0494 | 0.006209 | 80509 | 1 |  |
| 40s | 42 | 14132 | 663793 | 2782665 | 196.9053 | 0.006648 | 74150 | 1 |  |
| 40s | 45 | 14502 | 665300 | 2789858 | 192.3775 | 0.006195 | 79597 | 1 |  |
| 40s | 45 | 16029 | 795847 | 3381188 | 210.9419 | 0.006327 | 74440 | 1 |  |
| 40s | 47 | 16585 | 838662 | 3581116 | 215.925 | 0.005753 | 83076 | 1 |  |
| 50s | 51 | 15038 | 721200 | 3048857 | 202.7435 | 0.006379 | 77122 | 1 |  |
| 50s | 52 | 15245 | 716689 | 3036974 | 199.2112 | 0.00581 | 83024 | 1 |  |
| 50s | 52 | 17670 | 906992 | 3913673 | 221.4869 | 0.006168 | 76709 | 1 |  |
| 50s | 54 | 16700 | 844205 | 3612960 | 216.3449 | 0.006054 | 81328 | 1 |  |
| 50s | 57 | 16426 | 816719 | 3478521 | 211.7692 | 0.006054 | 80521 | 1 |  |
| 60s | 60 | 16450 | 826647 | 3539887 | 215.1907 | 0.00611 | 80539 | 1 |  |
| 60s | 63 | 17682 | 902752 | 3897550 | 220.4247 | 0.005775 | 82983 | 1 |  |
| 60s | 67 | 16847 | 845320 | 3615093 | 214.5838 | 0.006165 | 80148 | 1 |  |
| 60s | 67 | 17658 | 899469 | 3880938 | 219.7836 | 0.005957 | 81324 | 1 |  |
| 70s | 70 | 17590 | 896976 | 3863587 | 219.6468 | 0.005957 | 81967 | 1 |  |
| 70s | 70 | 17018 | 862627 | 3698093 | 217.3048 | 0.005798 | 82869 | 1 |  |
| 70s | 72 | 17185 | 872043 | 3747536 | 218.0702 | 0.006325 | 77231 | 1 |  |
| 70s | 72 | 17940 | 923820 | 3996863 | 222.7906 | 0.005906 | 82213 | 1 |  |
| 70s | 72 | 16571 | 820443 | 3502713 | 211.3761 | 0.005741 | 83398 | 1 |  |
| 70s | 73 | 16086 | 801417 | 3408898 | 211.9171 | 0.006195 | 79959 | 1 |  |
| 70s | 76 | 17505 | 898068 | 3866465 | 220.8778 | 0.005862 | 82733 | 1 |  |
| 80s | 81 | 17816 | 912590 | 3940394 | 221.1717 | 0.005838 | 81350 | 1 |  |
| 80s | 81 | 17479 | 890052 | 3829102 | 219.0687 | 0.005751 | 83209 | 1 |  |
| 80s | 81 | 16928 | 836459 | 3579732 | 211.4681 | 0.005827 | 82631 | 1 |  |
| 80s | 83 | 15032 | 724764 | 3058120 | 203.4406 | 0.006415 | 76590 | 1 |  |
